# Sonic Hedgehog Produced by Bone Marrow-Derived Mesenchymal Stromal Cells Supports Cell Survival in Myelodysplastic Syndrome

**DOI:** 10.1155/2015/957502

**Published:** 2015-03-15

**Authors:** Jixue Zou, Yan Hong, Yin Tong, Ju Wei, Youwen Qin, Shan Shao, Chun Wang, Kun Zhou

**Affiliations:** ^1^Department of Hematology, Shanghai Jiaotong University Affiliated First People's Hospital, Shanghai 200080, China; ^2^Experimental Research Center, Shanghai Jiaotong University Affiliated First People's Hospital, Shanghai 200080, China

## Abstract

The role of marrow microenvironment in the pathogenesis of myelodysplastic syndrome (MDS) remains controversial. Therefore, we studied the influence of bone marrow-derived mesenchymal stromal cells (BMSCs) from patients with different risk types of MDS on the survival of the MDS cell lines SKM-1 and MUTZ-1. We first demonstrated that the expression of Sonic hedgehog (Shh), smoothened (Smo), and glioma-associated oncogene homolog 1 (Gli1) was increased in MDS patients (*n* = 23); the increase in expression was positively correlated with the presence of high-risk factors. The Shh signaling inhibitor, cyclopamine, inhibited high-risk MDS BMSC-induced survival of SKM-1 and MUTZ-1 cells, suggesting a role for Shh signaling in MDS cell survival. Furthermore, cyclopamine-mediated inhibition of Shh signaling in SKM-1 and MUTZ-1 cells resulted in decreased DNMT1 expression and cell survival; however, exogenous Shh peptide had the opposite effect, suggesting that Shh signaling could regulate the expression of DNMT1, thereby modulating cell survival in MDS. In addition, the apoptosis of SKM-1 and MUTZ-1 cell increased significantly when cultured with cyclopamine and a demethylation agent, 5-Aza-2′-deoxycytidine. These findings suggest that Shh signaling from BMSCs is important in the pathogenesis of MDS and could play a role in disease progression by modulating methylation.

## 1. Introduction

Myelodysplastic syndrome (MDS) comprises a heterogeneous group of hematological disorders characterized by ineffective hematopoiesis, enhanced bone marrow apoptosis, and increased risk of progression to acute myeloid leukemia (AML). Because of its clinical heterogeneity and an incomplete understanding of its pathogenesis, MDS is very difficult to treat effectively. Among various treatment strategies, hematopoietic stem cell transplantation (HSCT) is currently the only potentially curative therapy. However, few patients qualify as good candidates for HSCT. Targeting epigenetic pathways to reverse pathologic inactivation of suppressor genes is another therapeutic possibility for MDS. Despite these findings, many questions remain unresolved, including the exact mechanism of MDS pathophysiology. While apoptosis is prominent in marrow cells from patients with early-stage MDS, proliferation and apoptosis resistance characterize more advanced MDS [1, 2]. As such, the study of different mechanisms involved in neoplastic cell survival could provide insights into MDS pathogenesis and offer new targets for the prevention of MDS progression.

Accumulating evidence indicates that, in certain hematological disorders, the hematopoietic microenvironment plays an indispensable role in disease development. MSCs are cytogenetically abnormal in a significant proportion of MDS and AML patients [3], although the majority of BMSC cytogenetic aberrations were totally different from those observed in their hematopoietic counterparts in some cases [4]. This may indicate potential involvement of BMSC in the pathophysiology of MDS/AML. Both the human bone marrow stromal cell line HS-5 and the murine stromal cell line MS-5 improved AML cell survival and attenuated apoptosis* in vitro* [5, 6]. The marrow microenvironment in MDS also has an increasingly recognized role in disease progression. Although apoptosis is markedly increased in MDS marrow cells, whether MDS marrow stromal layers have an inhibitory effect on the survival of hematopoietic cells is still controversial. It has been shown that MDS hematopoietic precursors tended to grow more colonies propagated on stromal layers from MDS marrow than on stromal layers from normal marrow [7]. However, in a recent study, a reduced ability of MDS bone marrow-derived mesenchymal stromal cells (BMSCs) to support CD34^+^ hematopoietic stem and progenitor cells (HSPC) was observed in long-term culture-initiating cell assays due to structural, epigenetic, and functional abnormalities in MDS BMSCs [8]. In addition to the unclear effect of BMSCs, the molecular mechanisms of interaction between MDS cells and their microenvironment remain incompletely defined.

Hedgehog (Hh) signals are critical for the proliferative regulation of hematopoietic progenitor cells. The Hh proteins share a common signaling pathway, and the transmembrane receptor patched 1 (Ptch1) binds to smoothened (Smo) in the absence of the Hh ligand. In the presence of the Hh ligand, Ptch1-mediated suppression of Smo is removed, and transcription factors glioma-associated oncogene homologs (Gli1, Gli2, and Gli3), as well as downstream target genes, are activated [9]. Components of the Hh pathway are expressed in erythroblasts in spleen and bone marrow and desert hedgehog (Dhh) influences early erythropoiesis and progenitor differentiation [10]. It was demonstrated that expression of the intrinsic Hh-signaling inhibitor, human Hh-interacting protein (HHIP), was reduced in AML/MDS-derived stromal cells and the reduction potentially correlates with the proliferation of leukemic cells [11]. Proliferation of primitive human hematopoietic cells induced by cytokines could be inhibited with the use of antibodies to hedgehog, and exogenous Sonic hedgehog (Shh) treatment induced the expansion of pluripotent human hematopoietic repopulating cells detected in immunodeficient mice [12]. Accumulating evidence suggests that altered activation of Shh signaling is implicated in the initiation, progression, and maintenance of various forms of cancer [13, 14].

However, the role of Shh in the pathogenesis of MDS and the contribution of the MDS bone marrow microenvironment to MDS progression is still unknown. Therefore, we studied the role of primary BMSCs from MDS patients on the survival of MDS cell lines SKM-1 and MUTZ-1. The results demonstrate that high-risk MDS BMSCs could promote the proliferation of SKM-1 and MUTZ-1. We specifically focused on the role of BMSC-induced Shh signaling in protecting MDS cells from apoptosis. In addition, we observed an association between increased Shh, Gli1, Smo, and DNA methyltransferase 1 (DNMT1) expression in BMSC-supported MDS blasts* in vitro*, suggesting an important role for Shh signaling in the survival advantage of MDS cells, which may provide novel molecular targets for therapeutic intervention.

## 2. Materials and Methods

### 2.1. Patients

Bone marrow samples were obtained from 23 untreated adult MDS patients (Table 1), 9 post-MDS AML patients (Table 2) at time of diagnosis, and 9 healthy donors. MDS patients (18–73 years, median age 56 years) were diagnosed by the World Health Organization (WHO) [15] and classified according to different international prognostic scoring system (IPSS) risk types [16] (low-risk, *n* = 12; intermediate (int), *n* = 6; high-risk, *n* = 5). As controls, normal bone marrow samples were obtained from 9 hematological normal bone marrow transplant donors (20–43 years old). All bone marrow samples were obtained from the Shanghai Jiaotong University Affiliated Shanghai First People's Hospital (Shanghai, China), between March 2013 and November 2014. Patient exclusion criteria were concomitant or previous cancer. In addition, informed consent was obtained from each subject before the bone marrow procedure, and the study was approved by the local ethics committee.

### 2.2. Isolation and Culture of Human Bone Marrow Stromal Cells and CD34^+^ Cells

Bone marrow mononuclear cells, separated from both normal and MDS bone marrow samples by Ficoll density centrifugation, were cultured in Dulbecco's modified Eagle's low-glucose medium (Gibco, Life Technologies, USA) with 20% heat-inactivated fetal bovine serum (FBS) (Gibco, Life Technologies) at 37°C in humidified atmosphere containing 5% CO_2_. After culturing for 24 h, nonadherent cells were removed and medium was changed every 3 days. Adherent cells were trypsinized and further expanded until an 80–90% confluence was reached. Cells between passages 3 and 5 were used. These BMSCs were >95% viable, as determined by trypan blue exclusion, and cell analysis was performed after the third passage. We established the purity of BMSCs layers in all samples by cell surface analysis using flow cytometry. Antibodies used for the identification of BMSCs included FITC-conjugated CD29, CD90, phycoerythrin- (PE-) conjugated CD105 and CD34, PC5-conjugated CD45, and ECD-conjugated CD14 (Biolegend, San Diego, CA).

CD34^+^ cells were isolated by magnetic-activated cell sorting (MACS) using MACS Direct CD34 Progenitor Cell Isolation Kit (Miltenyi Biotec, Bergish-Gladbach, Germany) following the manufacturer's protocol. The purity of the cell separation was established by staining with fluorescent-labeled anti-CD34 antibodies and CD34^+^ cell quantification by flow cytometric analysis. The purity of CD34^+^ populations was >95%, as determined by fluorescence-activated cell sorting analysis.

### 2.3. Cell Lines and Reagents

The SKM-1 cell line was derived from a patient with monoblastic leukemia following MDS [17] and was generously provided by the Institute of Hematology of Huazhong University of Science and Technology (Wuhan, China). MUTZ-1 cells were derived from a patient with MDS subtype refractory anemia with excess of blasts (RAEB) [18] and were kindly provided by the Institute of Hematology of Zhejiang University (Hangzhou, China).

The two human MDS cell lines were maintained in RPMI-1640 and IMDM medium (Gibco, Life Technologies, USA), respectively, with 10% fetal bovine serum (Gibco, Life Technologies) and 1% penicillin-streptomycin (Gibco, Life Technologies) and kept in a 5% CO_2_ incubator at 37°C.

Cyclopamine dissolved in DMSO (Sigma-Aldrich) was purchased from Tocris, R&D Systems, USA and Canada. Shh-N dissolved in phosphate-buffered saline was obtained from R&D Systems, USA. The control consisted of the same volume of relevant solvent alone. 5-Aza-2′-deoxycytidine (5-aza-dC) dissolved in DMSO was purchased from Sigma-Aldrich, USA.

### 2.4. Coculture

We incubated confluent BMSCs overnight prior to establishment of the cocultures. The total number of 1 × 10^4^ MUTZ-1 or SKM-1 cells was layered on a confluent layer of BMSCs derived from either normal donors or MDS patients with or without a 0.45-M Millipore membrane, on a 24-well plate for 72 h. Experiments were performed in triplicate using MUTZ-1 and SKM-1 cells cultured in medium alone as controls.

### 2.5. Cell Viability Assay

Cell viability was evaluated using CCK-8 (Beyotime). Cells (1 × 10^5^/mL) were seeded into 96-well plates in triplicate alone or with 10, 20, and 40 *μ*M cyclopamine. After 48 h of incubation, 10 *μ*L of CCK-8 was added to each well. Four hours later, absorbance was read at 450 nm using a microplate reader (Bio-Rad). Background absorbance was measured in wells containing only dye solution and culture medium. Data presented are the values subtracting background absorbance from total absorbance. The means of the triplicates were calculated.

### 2.6. Apoptosis Assays

Apoptosis assays were performed by flow cytometry using Annexin V-FITC Apoptosis Detection assay kit I (BD Pharmingen, Lot 25015). Cells were washed with cold PBS and resuspended in cold binding buffer containing FITC-conjugated Annexin V and PI. The mixture was incubated at room temperature in the dark for 15 min and was adjusted to the total volume of 500 *μ*L with Annexin V-binding buffer. The number of stained cells was immediately detected and analyzed using a flow cytometer (BD FACScan). The percentage of early and late apoptotic cells in each group was defined as positive for Annexin V-FITC but negative for PI staining and positive for both Annexin V-FITC and PI staining, respectively.

### 2.7. RT-PCR

Total RNA was isolated from cell lines and clinical samples using TRIzol reagent (Invitrogen Life Technologies, Carlsbad, CA, USA) following the manufacturer's instructions. Total RNA (1 *μ*g) was reverse-transcribed using an RNA Reverse Transcription kit (Applied Biosystems, Foster City, CA, USA) according to the manufacturer's protocols. Subsequent PCR amplification was performed using StepOnePlus Real-Time PCR System (Applied Biosystems) in a total volume of 20 *μ*L with the IQ SYBR Green Supermix (Bio-Rad laboratories) under the following conditions: 95°C for 10 min, followed by 40 cycles of 95°C for 15 s and 60°C for 10 min. The mRNA expression levels were normalized to expression of *β*-actin (ACTB) housekeeping gene. Gene expression relative to samples from healthy controls was calculated using the 2^−ΔΔCt^ method. Each assay was performed in triplicate. Primer sequences are provided in Table 3. PCR analysis of 21 MDS samples in 23 cases was performed because of the limited availability of specimens collected from the patients.

### 2.8. Immunofluorescence

Cells were fixed in 4% paraformaldehyde for 20 min at room temperature, then washed twice with PBS, and permeabilized with 0.25% Triton X-100. After incubation with 5% BSA for 1 h to block the nonspecific antibody binding, cells were stained with rabbit monoclonal anti-Shh (IgG1; 1 : 500; Santa Cruz Biotechnology) or anti-Gli1 (IgG; 1 : 500; Abcam) antibodies at 4°C for 10 h. Following three times washing in PBS for 5 min, the cells were incubated at 37°C for 1 h with Alexa Fluor 488-conjugated donkey anti-rabbit, IgG (1 : 200; Invitrogen Life Technologies) secondary antibodies. The preparations were then washed with PBS three times for 5 min each in the dark. Nuclei were stained with DAPI (Invitrogen) for 5 min. Immunofluorescence images were obtained using a Leica TCS SP8 laser scanning confocal microscope (Leica, Mannheim, Germany).

### 2.9. Statistical Analysis

All assays were performed in triplicate and data presented as the mean ± standard error. Student's *t*-test was performed for comparisons between two groups. One-way ANOVA (analysis of variance) was used for multiple comparisons between groups. Statistical analysis was conducted using SPSS 13.0. A value of *P* < 0.05 was considered statistically significant.

## 3. Results

### 3.1. Activation of Shh Signaling in MDS Cases

**Figure 1:**
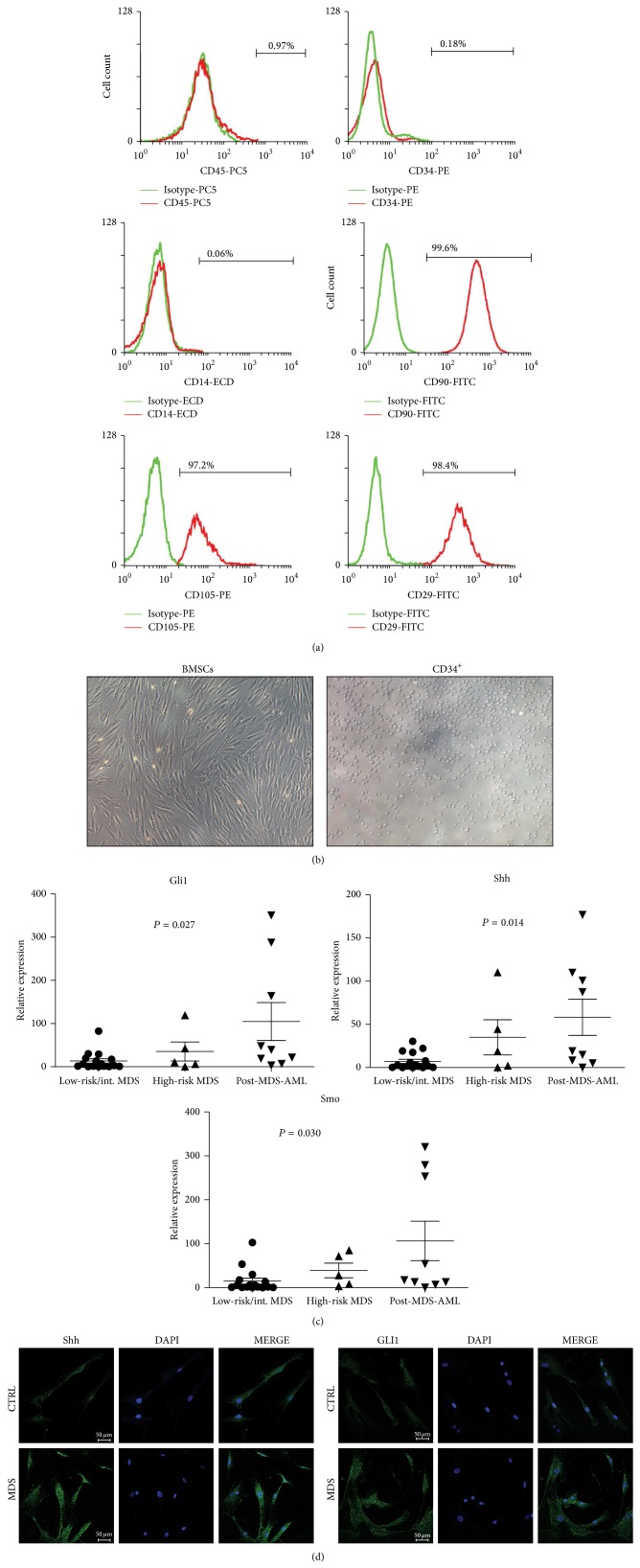
Activation of Shh signaling in MDS. Mean fluorescence intensity (MFI) of CD34, CD45, CD14, CD90, CD29, and CD105 was determined in BMSCs of MDS patients and healthy donors by flow cytometric analysis. Representative histograms are shown. Green line represents the respective isotype control and red line represents MDS-derived BMSCs (a). Representative micrographs of primary BMSCs and CD34^+^ stem cells ((b), ×200). Real-time PCR analysis of Gli1, Shh, and Smo in primary BMSCs (*n* = 30) from MDS and post-MDS AML marrow samples (c). Immunofluorescence images of SHH and GLI1 protein in primary bone marrow-derived mesenchymal stromal cells from MDS patients and healthy donors (CTRL) are shown (d).

Purified BMSCs obtained from both normal and MDS patients had a similar phenotype. Over 98% of BMSCs lacked expression of hematopoietic antigens CD14, CD34, and CD45 but were positive for BMSCs markers CD29, CD90, and CD105 (Figure 1(a)). Thus, the phenotype was consistent with the BMSCs criteria defined by the International Society for Cellular Therapy [19, 20]. Human bone marrow derived mesenchymal stromal cells showed fibroblast-like morphology (Figure 1(b)).

Real-time PCR analysis of Shh, Smo, and Gli1 in MDS and post-MDS AML patients showed that three essential components of the Shh pathway were higher in MDS BMSCs than in normal samples. Furthermore, expression levels positively correlated with MDS risks (Figure 1(c), *P* < 0.05). Both SHH and GLI1 proteins were overexpressed in high-risk MDS BMSCs (Figure 1(d)). These results indicate that upregulation of Shh signaling expression in primary MDS cells positively correlated with different MDS risk types and could be implicated in disease progression.

### 3.2. Inhibition of Shh Signaling Prevents BMSC-Induced MUTZ-1 and SKM-1 Cell Survival

**Figure 2:**
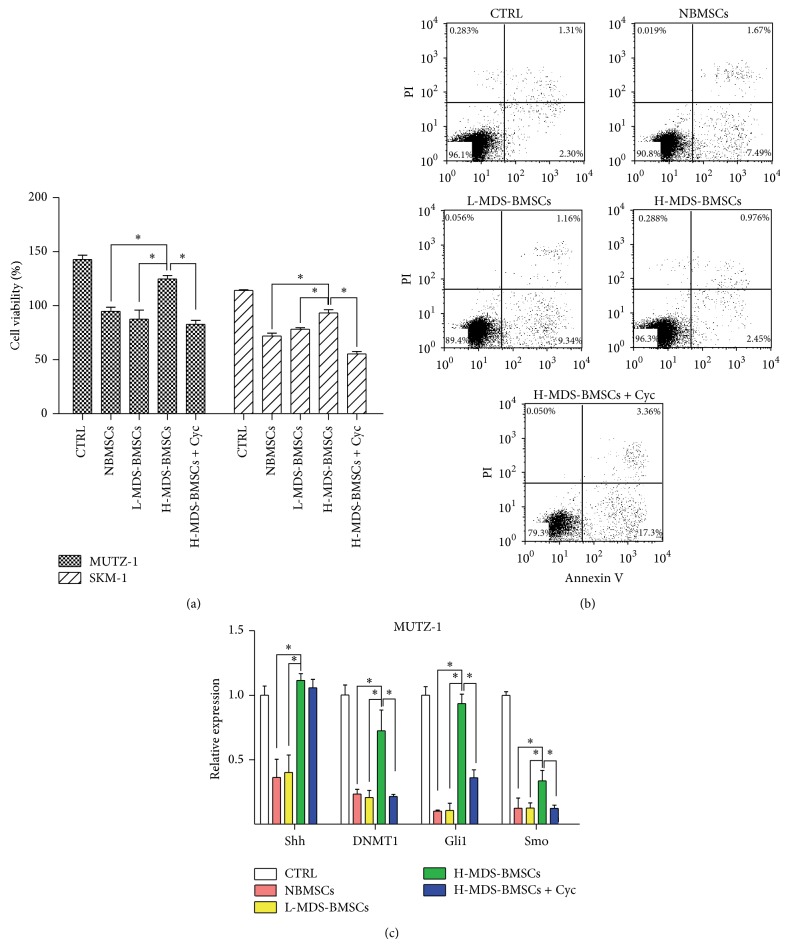
Inhibition of Shh signaling prevents BMSC-induced MUTZ-1 and SKM-1 cell survival. Proliferation and representative flow cytometric profile of MUTZ-1 cells cultured in the presence of control medium (CTRL) or BMSCs from normal (NBMSCs)/low-risk (L-MDS-BMSCs)/high-risk (H-MDS-BMSCs) MDS cases or high-risk MDS BMSCs with cyclopamine (Cyc) were determined by CCK8 assay (a) and Annexin V-PI assay (b). Expression of Shh, DNMT1, Gli1, and Smo was determined by real-time PCR (c). ^*^ *P* < 0.05. Data shown are from three independent experiments done in triplicate. Results are expressed as mean ± SEM.

To determine the influence of stromal cells in the microenvironment, MUTZ-1 and SKM-1 cells were cultured on bone marrow stromal cells for 72 h. BMSCs layers from both normal and MDS patients were able to inhibit the proliferation of MUTZ-1 and SKM-1 cells compared to the cells cultured with medium alone. However, it is noteworthy that the influence of BMSCs from high-risk MDS patients was evidently lower than that of BMSCs from normal and low-risk patients (Figure 2(a)). Consistently, the apoptosis of MUTZ-1 cells was 3-fold and 4-fold (2.3% to 7.2%; 2.3% to 8.9%) higher when cultured with BMSCs from healthy donors and low-risk MDS patients, respectively, than when the cells were cultured alone (Figure 2(b)). However, apoptosis of MUTZ-1 cells cultured with BMSCs from high-risk MDS patients was significantly lower than when the cells were cultured with BMSCs from healthy donors and low-risk MDS patients (*P* < 0.05). The influences of BMSCs and cyclopamine on the apoptosis of SKM-1 cells were particularly similar (data not shown). These results show the positive influence of a stromal microenvironment from high-risk MDS patients on the survival of MUTZ-1 and SKM-1 cells.

Furthermore, to confirm the contribution of Shh signaling to BMSC-induced MUTZ-1 and SKM-1 cell survival, MDS cell lines were similarly cultured on BMSCs in the presence of cyclopamine, an inhibitor of Hh signaling. The proliferation was 41% lower (82.7 ± 3.8% to 124.8 ± 3.1%) and the apoptosis of MUTZ-1 cells was 7 times higher when the cells were cultured in the presence of cyclopamine with high-risk MDS BMSCs (35% versus 5%) than when they were cultured on BMSCs alone (Figures 2(a) and 2(b)). As increased expression of Shh was observed in primary MDS BMSCs, these results confirmed that Shh mediated the increase in the survival of MUTZ-1 and SKM-1 cells grown on BMSCs.

Real-time PCR analysis was performed subsequently to observe the effects of BMSCs on Shh pathway components in MUTZ-1 and SKM-1 cells. Given the important role of DNA methylation in MDS pathogenesis [21], expression of DNMT1 was also determined. The expression of Shh, Gli1, and DNMT1 was significantly higher when MUTZ-1 cells were cultured on high-risk MDS BMSCs than when they were cultured on normal or low-risk MDS BMSCs (*P* < 0.05). Furthermore, when cyclopamine was added to high-risk MDS BMSCs, their influence on MDS cell lines was abrogated (Figure 2(c)). SKM-1 cells were similarly influenced by BMSCs and cyclopamine exposure (data not shown). These results clearly demonstrate the positive influence of Shh signaling mediated by BMSCs from high-risk MDS on survival of MUTZ-1 and SKM-1 cells. Together, these experiments indicate a role for the tumor microenvironment of high-risk MDS via BMSC-mediated Shh signaling in the survival of MDS cells.

### 3.3. Modulation of Shh Signaling Influences the Survival of MUTZ-1 and SKM-1

**Figure 3:**
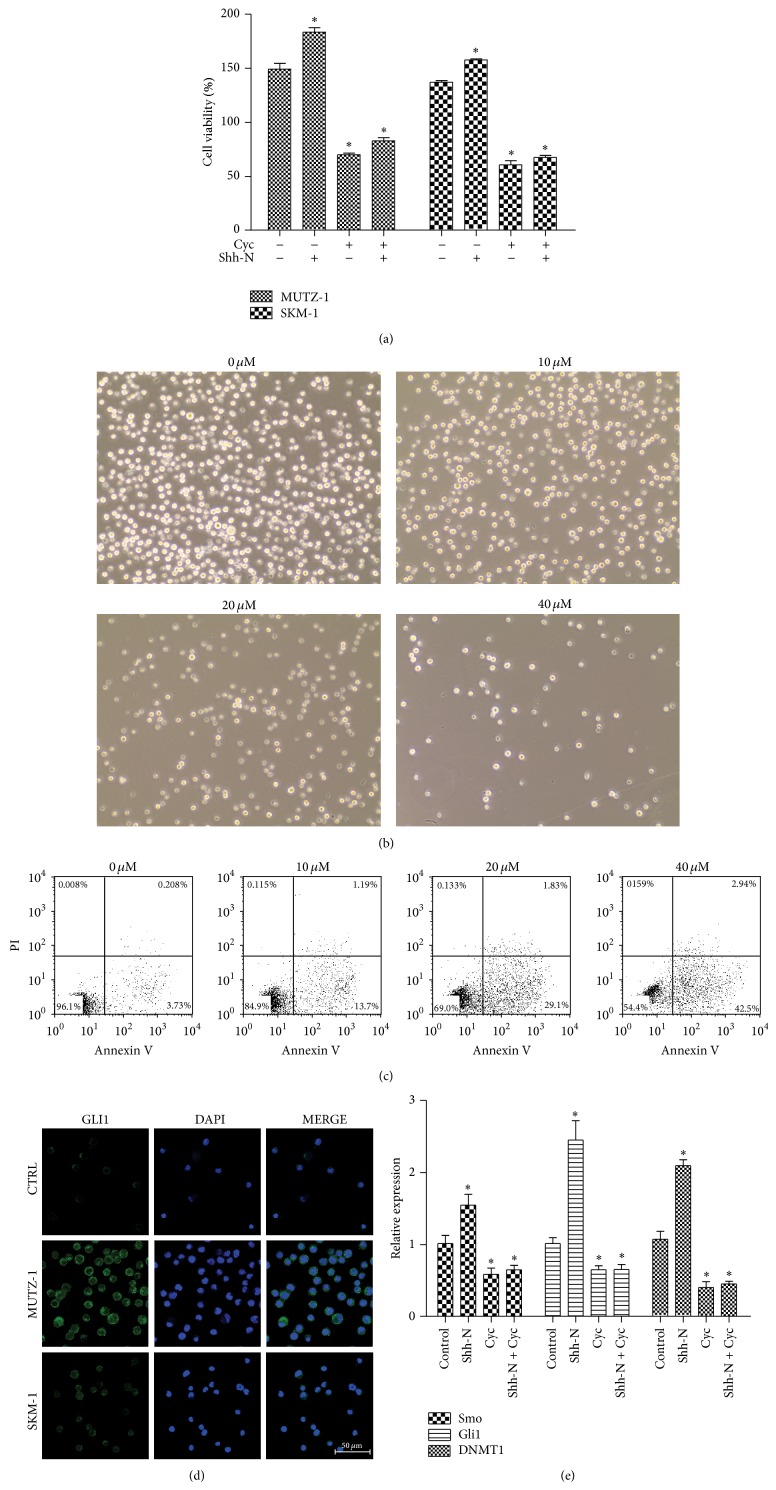
Modulation of Shh signaling influences the survival of MUTZ-1. MUTZ-1 cells were cultured in the presence of medium, exogenous Shh peptide (Shh-N) (500 ng/mL), cyclopamine (Cyc) (20 *μ*M), and Shh-N + cyclopamine for 48 h, and cell survival was determined by the CCK8 and Annexin V-PI assays. There was significantly increased proliferation of MUTZ-1 cells in the presence of exogenous Shh peptide and decreased cell proliferation in the presence of cyclopamine, as determined by the CCK8 assay (a). Cyclopamine at concentrations of 10, 20, and 40 *μ*M induced inhibition of cell proliferation ((b), ×200) and increased apoptosis (c) in MUTZ-1 cells within 48 h in a dose-dependent manner. Expression of GLI1 protein in CD34^+^ stem cells from healthy donors, MUTZ-1 cells, and SKM-1 cells shown by confocal laser scanning microscopy (d). Real-time PCR analysis of Smo, Gli1, and DNMT1 after treatment with Shh-N (500 ng/mL) and/or cyclopamine (20 *μ*M) for 72 h (e). Expression of beta-actin (ACTB) was used as a housekeeping control gene. Fold-change was calculated with 2^−ΔΔCt^ method compared with controls. Results are expressed as average ± SEM of triplicates each. ^*^ *P* < 0.05.

To determine the specific influence of Shh signaling on the survival of MUTZ-1 and SKM-1 cells, Shh signaling was modified* in vitro* by the addition of exogenous Shh peptide (Shh-N) and SMO inhibitor cyclopamine. The survival of MUTZ-1 cells was higher in the presence of Shh-N (183.4 ± 4.1% versus 149.2 ± 5.4%, *P* < 0.05) and significantly lower in the presence of Shh-N plus cyclopamine (82.9 ± 3.0%, *P* < 0.05) or cyclopamine alone (70.2 ± 1.4%, *P* < 0.05) compared to the survival when the MUTZ-1 cells were cultured alone (Figure 3(a)). Abrogation of Shh signaling with cyclopamine at concentrations of 10, 20, and 40 *μ*M led to survival inhibition of MUTZ-1 and SKM-1 cells in a dose-dependent manner in 48 h (Figure 3(b)). These results correlated with apoptosis data, supporting the interpretation of inhibited survival (Figure 3(c)). The results of SKM-1 cells were consistent with findings from MUTZ-1 cells.

Overexpression of the Shh signaling transcription factor GLI1 protein was also observed in MUTZ-1 and SKM-1 cells (Figure 3(d)). In addition, the effects of cyclopamine on DNMT1 and the Smo downstream transcription factor Gli1 were determined using real-time PCR. The expression of DNMT1 and Gli1 in the presence of cyclopamine was lower than that in the controls, but it was higher in the presence of Shh-N (Figure 3(e)). Collectively, these experiments clearly indicate that modulation of Shh signaling in MUTZ-1 and SKM-1 cells influenced cell survival and DNMT1 expression. Thus, Shh signaling could be associated with DNA hypermethylation and represent an important factor in MDS pathogenesis.

### 3.4. Cyclopamine and Demethylating Agent 5-Aza-dC Synergize to Inhibit Survival of MUTZ-1 and SKM-1 Cells

**Figure 4:**
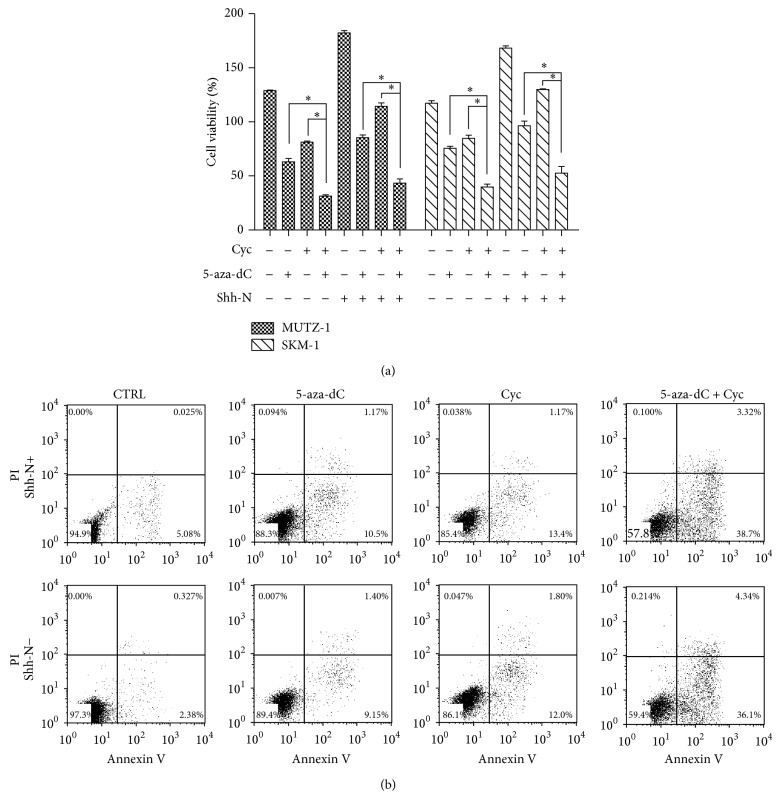
Cyclopamine and demethylating agent 5-aza-dC synergize to induce survival inhibition of MUTZ-1 and SKM-1. MUTZ-1 cells were treated with a combination of cyclopamine and 5-aza-dC or with either agent alone in the presence or absence of Shh-N for 48 h. Cell proliferation and apoptosis were determined using CCK8 assay (a) and Annexin V-PI staining analysis (b), respectively. ^*^ *P* < 0.05. Flow cytometric data are shown from one representative experiment.

To further investigate the effects of downregulation of Shh signal and demethylation on MDS cell survival, MUTZ-1 and SKM-1 cells were cultured in the presence of 5-aza-dC, an inhibitor of DNA methyltransferase, with or without cyclopamine or Shh-N. Inhibition of DNA methylation with 5-aza-dC in combination with cyclopamine resulted in a synergistic inhibition of MUTZ-1 and SKM-1 cell proliferation (Figure 4(a)). The proliferation of MUTZ-1 cells was significantly lower in the presence of cyclopamine and 5-aza-dC than in the presence of 5-aza-dC alone (31.5 ± 1.0% versus 63.0 ± 3.0%, *P* < 0.05). To confirm the synergistic interaction of Shh signaling inhibition and demethylation, we also used flow cytometry to analyze apoptosis. Similarly, in the presence or absence of Shh-N, apoptosis of MUTZ-1 cells was significantly higher following treatment with both agents (38.9% and 36.4%) compared with that in cells treated with either 5-aza-dC alone (15.1% and 10.8%, *P* < 0.05) or cyclopamine alone (12.9% and 7.1%, *P* < 0.05) (Figure 4(b)). Together, these results indicate that inhibition of Shh signaling synergistically decreased MDS cell proliferation and increased apoptosis when combined with 5-aza-dC.

## 4. Discussion

Myelodysplastic syndromes (MDSs) are clonal disorders of hematopoietic stem cells (HSCs). As the underlying mechanisms and critical regulatory molecules of the hematopoietic microenvironment are unclear, the development of effective therapy for MDSs was difficult. Our work showed that the expression of Shh signaling pathway components such as Shh, Gli1, and Smo in primary MDS stromal cells (*n* = 23) was significantly increased and positively correlated with IPSS risk types of MDS. Inhibition of Shh signaling reduced the proliferation of MUTZ-1 and SKM-1 cells supported by high-risk MDS stromal cells through blocking DNMT1. This result suggested the SHH-GLI1-DNMT1 pathway could be a potential therapeutic target.

MDS is a heterogeneous disease and has a high risk of progression to AML. Expression of AML1 mutants induced MDS/AML of distinct phenotypes in mice [22]. However, the pathogenesis and progression of MDS remain unclear. Insufficient hematopoiesis is the cause of most morbidity in patients with low-risk MDS. Geyh et al. had showed that the MDS-derived BMSCs could not effectively support CD34^+^ HSPC in long-term culture-initiating cell assays [8]. Consistent with Geyh's study, we also found that normal and low-risk MDS-derived BMSCs inhibited MDS cell proliferation. We further discovered that the inhibition was alleviated as the risk of disease increased. High-risk MDS BMSCs promoted the proliferation of SKM-1 and MUTZ-1 cells, whereas normal and low-risk MDS BMSCs inhibited their proliferation. Increased stromal expression of Shh, Gli1, and Smo was also observed in primary MDS cells. Their expression level positively correlated with MDS risks and was much lower than in post-MDS AML. It was previously suggested that, in human myeloid leukemia (both AML and CML), stromal elements were functionally impaired [23]. According to the experiments, the abnormal microenvironment seemed to participate in the progression of the disease, since it contributed to the selective expansion of the malignant clone by favoring proliferation of neoplastic cells and inhibiting the growth of normal stem and progenitor cells [24]. These reports suggested that stromal dysfunction could be an early and indispensable event in MDS development. Shh signaling was more active in advanced stages of MDS and could play an important role in MDS progression to AML.

The precise molecular mechanism of how stromal dysfunction contributes to the proliferation of MDS or leukemic blasts remains unclear. Kobune et al. demonstrated that stromal HHIP expression was decreased in AML/MDS-derived stromal cells [11]. Aberrant stromal HHIP reduction could contribute to the progression of AML/MDS. Another preliminary study indicated that cyclopamine could dramatically restore multidrug resistance and inhibit AML cells* in vitro* [25]. It was demonstrated that SHH and GLI1 are expressed in leukemic cell lines and primary leukemic blasts [26, 27], and recent evidence suggests that inhibitors of the Hh pathway could be effective in reverting leukemia chemoresistance [28]. In this regard, we demonstrated that inhibiting Hh signaling with cyclopamine decreased high-risk MDS BMSC-induced MUTZ-1 and SKM-1 cell survival. As increased expression of Shh was observed in primary MDS BMSCs, these results indicate that high-risk MDS BMSCs could promote neoplastic cell proliferation by actively promoting indispensable Shh signaling. Moreover, this further confirmed that Shh signaling deregulation was an important mechanism in MDS pathogenesis and that abnormal BMSCs participated in this process.

However, the mechanisms by which stromal Shh signaling from high-risk MDS influenced MDS blasts remain unclear. However, it is notable that, in our study, DNMT1 was also upregulated in MDS cell lines and that cyclopamine downregulated its expression. As the functional inhibitor we used will influence signaling from all three hedgehog proteins (Sonic, desert, and Indian hedgehog), we used exogenous Shh peptide which could revert the effects of SMO inhibitor cyclopamine on the survival of MDS cells (Figure 3(a)). These findings suggest that Shh signaling blockade alone or in combination with demethylating treatments could potentially represent a novel intervention for MDS. Although demethylating agents are currently widely used in MDS treatment, their therapeutic effect is limited. To investigate whether inhibition of DNA methylation coupled with blockade Shh signaling pathway could enhance the induction of MDS cell apoptosis, we treated MDS cells with 5-aza-dC in combination with cyclopamine. We demonstrated for the first time that cyclopamine acts in concert with 5-aza-dC to synergistically induce MDS cell apoptosis. These results suggest that Shh signaling and abnormal DNA methylation participate in the progression through independent but interrelated mechanisms of MDS pathogenesis. Clinical-grade SMO inhibitors are currently under clinical trials for both solid tumors and hematologic malignancies [29, 30]. Targeting these interactions could provide a more effective alternative therapeutic strategy, either alone or in combination, compared with conventional treatments.

Our study demonstrated that active Shh signaling from high-risk MDS stromal cells promoted the proliferation of MDS cell lines. Downregulation of Shh signaling reverted the inhibition of MUTZ-1 and SKM-1 cell apoptosis by high-risk MDS BMSCs. Moreover, cyclopamine synergistically induced MDS cell apoptosis with demethylating agent 5-aza-dC, indicating that Shh signaling could be associated with DNA hypermethylation and represent an important aspect of in MDS pathogenesis.

## Figures and Tables

**Table 1:** The characteristics of myelodysplastic syndrome patients.

Number	Diagnosis	Age/sex	Marrow blasts (%)	Karyotype	IPSS
(1)	RA	39/F	0	46,XX[16]	0
(2)	RA	70/M	0	46,XY[20]	0
(3)	RA	54/F	2	46,XX[5]	0
(4)	RARS	65/F	2	46,XX[22]	0
(5)	RA	61/M	0	46,XY[4]	0
(6)	RARS	65/M	3	46,XY[7]	0
(7)	RA	18/F	0	46,XX[7]	0
(8)	RA	69/F	0	46,XX[16]	0
(9)	RCMD	30/M	0	46,XY,add(7)(q33),del(21)(q22)[10]/46,XY[10]	1.5
(10)	RCMD	21/M	1	46,XY[20]	0.5
(11)	RCMD	61/F	0	46,XX[6]	0.5
(12)	RCMD	56/M	2	45,X,−Y[12]/46,XY[3]	0.5
(13)	RAEB1	56/M	7	46,XY[8]	1.0
(14)	RCMD	62/M	3	46,XY,del(1)(p12p32)[6]/47,idem, +del(1)(p12p32)[4]/46,XY(10)	1.0
(15)	RAEB1	62/F	8	47,XX,+8[15]/46,XX[3]	1.5
(16)	RCMD	69/M	2	46,XY[6]	0.5
(17)	RCMD	38/M	2	46,XY[11]	0.5
(18)	RAEB1	73/M	7	47,XY,+8[25]	1.0
(19)	RAEB2	61/M	17	47,XY,+8[15]/46,XY[10]	2.5
(20)	RAEB2	43/F	11	46,XX,inv(3)(q21q26)[16]/46,XX[4]	2.5
(21)	RAEB2	73/M	18	46,XY,+8[12]/46,XY[3]	2.5
(22)	RAEB2	71/M	12	45,XY,−7[8]/46,XY,	3.0
(23)	RAEB2	64/M	16	46,XY,del(5)(q31,q34)[9]/46,XY[5]	2.5

F and M indicate female and male; IPSS, International Prognostic Scoring System; RA, refractory anemia; RARS, refractory anemia with ringed sideroblasts; RCMD, refractory cytopenia with multilineage dysplasia; RAEB, RA with excess blasts.

**Table 2:** The characteristics of postmyelodysplasia acute myeloid leukemia patients.

Number	Age/sex	Marrow blasts (%)	Karyotype
(1)	73/M	21	46,XY[10]
(2)	60/F	31	46,XX[20]
(3)	69/F	69	46,XX[16]
(4)	30/M	35	46,XY,del(21)(q22)[10]
(5)	64/M	28	46,XY[8]
(6)	71/M	36	46,XY[7]
(7)	18/F	23	46,XX[12]
(8)	69/F	51	46,XX[19]
(9)	64/M	73	46,XY,del(5)(q31,q34)[6]

F and M indicate female and male.

**Table 3:** Primer sequences for real-time PCR.

Genes	Forward (5′-3′)	Reverse (5′-3′)
Shh	CGGAGCGAGGAAGGGAAAG	TTGGGGATAAACTGCTTGTA
Gli1	GGCTGGACCAGCTACATCAAC	TGGTACCGGTGTGGGACAA
Smo	ATCTCCACAGGAGAGACTGGTTCGG	AAAGTGGGCCTTGGGAACATG
DNMT1	AACCAACACCCAAACAGAAACT	CTCCATCTTCGTCCTCGTCAG
*β*-actin	CGTGCTGCTGACCGAGG	GAAGGTCTCAAACATGATCTGGGT
